# Estimating the cost of illness of non‐alcoholic fatty liver disease in Bangladesh

**DOI:** 10.1002/jgh3.12960

**Published:** 2023-09-04

**Authors:** Shahinul Alam, Md. Saiful Islam Alin, Farhana Begum, Shah Mohammad Fahim, Zareen Tasnim, Md. Mahabubul Alam

**Affiliations:** ^1^ Department of Hepatology BSMMU Dhaka Bangladesh; ^2^ University of Rajshahi Rajshahi Bangladesh; ^3^ icddr,b Dhaka Bangladesh; ^4^ Programme for Emerging Infections, Infectious Diseases Division icddr,b Bangladesh

**Keywords:** Bangladesh, cirrhosis, cost of illness, liver disease, NAFLD, NASH

## Abstract

**Background and Aim:**

Non‐alcoholic fatty liver disease (NAFLD) is a growing concern, affecting about 45 million of the Bangladeshi population. There is a paucity of research on the economic burden of NAFLD. The study aims to estimate the cost of illness of NAFLD in Bangladesh.

**Methods:**

In this prospective, cross‐sectional study, a total of 250 patients of NAFLD, non‐alcoholic steatohepatitis (NASH), and NASH cirrhosis were included from public and private hospitals. Costs of hospitalization, physician fees, investigation costs, expenditures on medical procedures, drugs; and nonmedical costs such as transport expenses and other informal payments (tips) were estimated.

**Results:**

The overall cost per patient per evaluation was (16.90–46 942.00) USD. The cost in public and private hospitals was 384.76 and 1146.93 USD, respectively. The cost per patient of NAFLD was 157.91 (16.90–955.08) USD, and for NASH cirrhosis was 1783.80 (422.48–46 942) USD. The cost of illness increased to USD 281.18 for diabetics and 254.52 USD for hypertensive. If all the NAFLD patients are evaluated once in healthcare settings, the projected cost will be 7.11 billion USD. In NAFLD, cost for investigations, medicines, transportation, and consultation of physicians was 49.08%, 32.41%, 11.11%, and 6.67%, respectively.

**Conclusions:**

NAFLD is causing a huge economic burden to the healthcare system. The cost of illness is increased with NASH cirrhosis. Overall, this study provides valuable insights into the economic burden of NAFLD in Bangladesh and emphasizes the several ways of intervention to reduce the cost by preventive measures and accessible healthcare for affected individuals.

## Graphical abstract

The cost per patient of NAFLD was 157.91 (16.90–955.08) USD, and for NASH cirrhosis was 1783.80 (422.48–46 942) USD. The cost of illness was increased to USD 281.18 for diabetics and 254.52 USD for hypertensive. If all the NAFLD patients are evaluated once in healthcare settings, the projected cost will be 7.11 billion USD.

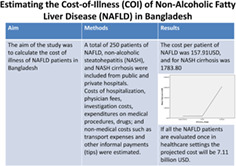

## Introduction

The spectrum of non‐alcoholic fatty liver diseases (NAFLD) is associated with a considerable socioeconomic burden, which along with its rising prevalence is a major public health challenge.^1^ NAFLD is a disorder originating from hepatic steatosis and continues to be the most common liver disease across the world. NAFLD can be established by the exclusion of other causes of hepatic fat accumulation, which includes unrestrained consumption of alcohol and alternative etiologies of liver diseases, such as chronic viral hepatitis. NAFLD is linked with many metabolic ailments, such as obesity, type II diabetes, dyslipidemia, and metabolic syndrome. It holds a significant prevalence across the globe; however, variations in genetic sequences, changing patterns of sedentary lifestyles, and improper nutrition can modify the load of NAFLD in distinct regions.^2^ About 45 million (33.86%) people in Bangladesh are affected by NAFLD.^3^ Non‐alcoholic steatohepatitis (NASH)—the inflammatory version of NAFLD, is established to be the hepatic adverse effect of metabolic syndrome. NASH is defined by changes in hepatic histological structure and includes steatosis, injury, and varying proportions of fibrosis. NASH may progress to NASH cirrhosis, hepatocellular carcinoma, or end‐stage liver disease, and ultimately liver transplantation remains the only treatment option.^4^, ^5^

Only morbidity and mortality associated with the disease are considered to measure disease burden, which reflects an incomplete picture. Few studies have been conducted to address the economic impacts of this disease around the world, especially in Asian countries. A recent study on the economic load estimated the annual burden associated with all incidents and prevalent NAFLD cases in the United States at $103 billion, which was calculated to be about €35 billion in Germany, France, Italy, and the United Kingdom.^6^ The cost of illness of NAFLD cirrhosis was also explored in Japan.^7^ About one‐third population of Bangladesh is affected by NAFLD,^3^ and the prevalence is higher than in surrounding nations, putting this population at an increased risk of liver‐related morbidity and mortality. Early to midlife adults, diabetic, overweight, and obese individuals, rural women, and married individuals were found to be at a greater risk of developing NAFLD than others.^3^, ^8^, ^9^ So far, it is sought for, from the Bangladeshi point of view, there is no evidence of pertinent data regarding the economic impact of NAFLD. Since the prevalence of NAFLD is significant and perhaps, rising in this country, it is imperative to estimate the economic burden of NAFLD among the affected population. The aim of the study was to calculate the cost of illness of NAFLD patients in Bangladesh. This type of analysis may assist in addressing the rising prevalence of this asymptomatic condition, which poses a major public health challenge.

## Methods

### Study design and sample

This observational, prospective, cross‐sectional study was conducted between March 2021 and February 2022. The study was done in two settings: Bangabandhu Sheikh Mujib Medical University (BSMMU), which is the largest, apex, multidisciplinary hospital in Bangladesh. To assess the cost of illness, this is the hospital of standard care at public sector of the country.

The second site was Ibn Sina Hospital, which is the largest, multidisciplinary, private hospital of the country established in 1983.It consists of 435 beds. It is a tertiary‐level hospital of a middle category that is approachable by all economic classes of the population and situated in Dhaka. Both the study sites were selected purposively.

A total of 250 respondents were included in the study: one hundred and fifty patients with the diagnosis of simple NAFLD, 50 patients with NASH, and 50 patients with NASH cirrhosis. Patients who received healthcare services from both the Out‐Patient Department (OPD) and In‐Patient Department (IPD) of the Hepatology Department of BSMMU and Ibn Sina Hospital respectively and during the study period comprised to be the study population. The study included patients with the diagnosis of NAFLD, NASH, and NASH cirrhosis in hospital records, had regular visits for check‐ups, and had been evaluated with relevant clinical, laboratory, and imaging tests. NASH was diagnosed with histological findings of NAFLD activity score ≥5. NASH cirrhosis was diagnosed with sonographic findings with clinical findings of stigmata of chronic liver disease/endoscopic findings of portal hypertension/ stiffness >14 kPa by fibroscan. Patients with a history of alcohol consumption, consumption of drugs causing fatty liver, and chronic liver disease due to any other cause were excluded from the study.

### Data collection and cost analysis

Written informed consent was obtained from each patient, and data were collected using a pre‐tested, structured questionnaire through face‐to‐face interviews. We have collected the receipt of medicine cost, hospital bill, laboratory bill, medical procedure bill, and consultation fee. Direct nonmedical costs were transportation, food, and others by interview only. The main aim was to estimate the cost of illness of NAFLD in Bangladesh. Intangible costs were not considered as they were difficult to calculate. The only costs that were considered were direct medical costs: costs of hospitalization, physicians' fee/visit, laboratory investigation, medical procedures, medicines; and direct nonmedical costs: transportation/travel costs, and other informal costs. The indirect cost/loss was not evaluated: productivity losses due to morbidity of the patient (absenteeism) and cost of the informal caregiver.

A micro‐costing bottom‐up method was used in order to estimate the costs of the direct medical and nonmedical components. Initially, each component per patient was quantified and unit costs were measured. After that, the total cost for a component was obtained by multiplying the unit cost and number of resources used or services taken by each patient (as reported through the questionnaire survey or by money receipt) within the last 3‐month time period or with a comprehensive evaluation in OPD/IPD. Price of investigation and diagnosis (various liver function tests), medical procedures (endoscopy, biopsy, and variceal ligation), drugs, hospitalization fee, and physician visit were included as the components of direct medical components. The components of the direct nonmedical costs were transportation costs of the patients and other informal costs incurred in order to receive health care. The costs attributed to nonmedical components were noted from the filled‐up questionnaires. Afterward, costs for each category (IPD fees, OPD visits, diagnosis tests, procedural examinations and drugs, travel costs, and other costs) were added to determine the total cost per person in 3 months. The cost was calculated in Bangladeshi Taka and then converted to USD at a rate of 1 USD = 106.51 BDT.^10^

### Statistical analysis

All data were analyzed using Statistical Package for Social Science (SPSS) Version 26. Data were presented as tables, bar charts, and line diagrams. The results are presented as mean ± SD, median, and range for the quantitative data and as numbers or percentages for the categorical or qualitative data. The statistical differences in the quantitative data were assessed using *t*‐test, Mann–Whitney *U* test, and ANOVA test. For all of the tests, significance was achieved at *P* < 0.05.

## Results

### Sociodemographic characteristics of the respondents

Table 1 shows the distribution of sociodemographic characteristics of the patients. The mean age ± SD was 43.78 ± 11.78 years. In terms of gender, male and female patients were 154 and 96 in number, respectively. Rural and urban residents were 157 (62.8%) and 93 (37.2%). An equal number of respondents were from public and private settings, making it a total of 250. Regarding academic status, most of the respondents were bachelor and above (25.2%) or secondary level (24.4%). As per occupational status, the majority of patients were housewives (34.8%) or others (22%). Regarding monthly income, the mean ± SD was 216 ± 143.55 USD. The mean ± SD of BMI was 26.40 ± 4.29 kg/m^2^. According to the BMI category, most patients were overweight (48.8%) or normal (35.5%). Among the participants, 61 (37.4%) and 62 (38%) had diabetes and hypertension as co‐morbidity, respectively. All of the study population was negative for HBsAg, Anti Hbc, and Anti HCV. None of them have taken alcohol in their lifetime. Among the respondents, 150 were NAFLD, followed by 50 patients each with NASH and NASH cirrhosis. The median total cost per patient per evaluation was 184.48 USD.

**Table 1:** Distribution of sociodemographic characteristics of the respondents (*n* = 250).

Characteristics	Values
Age [in years] (mean ± SD)	43.78 ± 11.78
Sex Male/Female (*n*/%)	154 (61.6)/96 (38.4)
Place of residence Urban/Rural (*n*/%)	93 (37.2)/157 (62.8)
Hospital type (public/private)	125/125
Academic status (*n*/%) Bachelor and above Higher Secondary Secondary Primary Illiterate	63 (25.2) 22 (8.8) 61 (24.4) 57 (22.8) 47 (18.8)
Occupation (*n*/%) Housewife Others Service Business Farmer	87(34.8) 55 (22) 53 (21.2) 35 (14) 5 (2)
Monthly income [USD] (Mean ± SD)	216 ± 143.55
BMI [kg/m^2^] (Mean ± SD)	26.40 ± 4.29
BMI Category (*n*/%) Underweight Normal Overweight Obese	8 (3.3) 94 (35.5) 118 (48.8) 30 (12)
Disease stage (*n*/%) NAFLD NASH NASH cirrhosis	150 (60) 50 (20) 50 (20)

BMI, body mass index; NAFLD, non‐alcoholic fatty liver disease; NASH, non‐alcoholic steatohepatitis; USD, United States Dollar.

### Cost analysis of the respondents

Table 2 demonstrates the distribution of total costs for disease category and hospital settings of the respondents. The overall cost per patient was 184.48 (16.90–46 942) USD. In addition to this, both diabetes and hypertension demonstrated an increasing cost of 281.18 (21.97–46 942.00) and 254.52 (23.94–46 942.00) USD. Single‐time comprehensive evaluation at the public and private hospitals cost USD 148.15 (16.90–3098.17) and 212.18 (16.90–46 942), respectively. Regarding NAFLD, the cost was 157.91 (16.90–955.08) USD. In the case of public hospital settings, the cost was 138.95 (16.90–609.80) USD, whereas in private settings, it is 190.87 (49.76–955.08) USD.

**Table 2:** Distribution of total expense for disease category and hospital settings of the respondents.

Disease category	Overall [Median and range] [USD]	Public [Median and range] [USD]	Private [Median and range] [USD]	*P*‐value
**NAFLD**				
Median (Range)	157.91 (16.90–955.08)	138.95 (16.90–609.80)	190.87 (49.76–955.08)	0.001*
**NASH**				
Median (Range)	117.59 (16.90–1032.54)	64.78 (16.90–455.62)	170.45 (47.32–1032.54)	0.000*
**NASH cirrhosis**				
Median (Range)	1783.80 (422.48–6942)	938.84 (422.48–3098.17)	3004.29 (478.81–46 942)	0.000*
**Diabetes Mellitus**				
Median (Range)	281.18 (21.97–46 942)	79.43 (21.97–186.17)	332.19 (67.22–46 942)	0.000*
**Hypertension**				
Median (Range)	254.52 (23.94–46 942)	111.06 (23.94–938.84)	281.18 (70.98–46 942)	0.013*
**Total**				
Median (Range)	184.48 (16.90–46 942)	148.15 (16.90–3098.17)	212.18 (16.90–46 942)	0.003*

* Statistically significant.

NAFLD, non‐alcoholic fatty liver disease; NASH, non‐alcoholic steatohepatitis; USD, United States Dollar.

With context to NASH, the cost per patient was 117.59 (16.90–1032.54); in public hospitals, it was 64.78 (16.90–455.62), and in private hospitals, it was 170.45 (47.32–1032.54). The differences in expenses between NAFLD and NASH were not statistically significant. With regard to NASH cirrhosis, the median expense was much higher than the previous two categories of disease, which amounted to 1783.80 (422.48–46 942) USD, in the public hospital 938.84 (422.48–3098.17) and in a private hospital (478.81–46 942) USD. In this case, the cirrhosis cost at the private hospital was 3.2 times higher than that of the public hospital. In the case of NAFLD and NASH, expenditure at a private hospital was 1.38 and 2.63 times higher than that of the public hospital.

About 45 million people have been suffering from NAFLD in Bangladesh.^11^ If all the NAFLD patients are evaluated once in healthcare settings, the projected expenditure will be 7.11 billion USD. In the natural course, the aggressive form is NASH, which may lead to liver cirrhosis and hepatocellular carcinoma. Assuming only 1% of individuals with developing NASH progress to cirrhosis, the cost of a single comprehensive evaluation/person would be 1783.80 USD. The projected total cost will be 802.35 million USD.^12^

**Figure 1:**
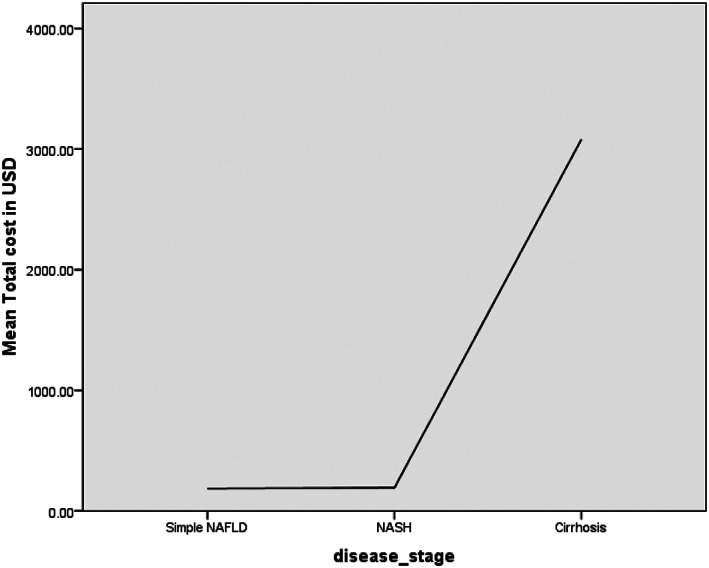
Demonstration of the mean of the total cost as per the stage of disease.

Figure 1 above shows a line diagram demonstrating the mean of total expenditure per stage of the disease. Simple NAFLD and NASH show similar expenses in terms of the mean total cost, which is 190.68 USD. However, for NASH cirrhosis, the mean total expenditure has risen highly, which is 3146.15 USD.

**Figure 2:**
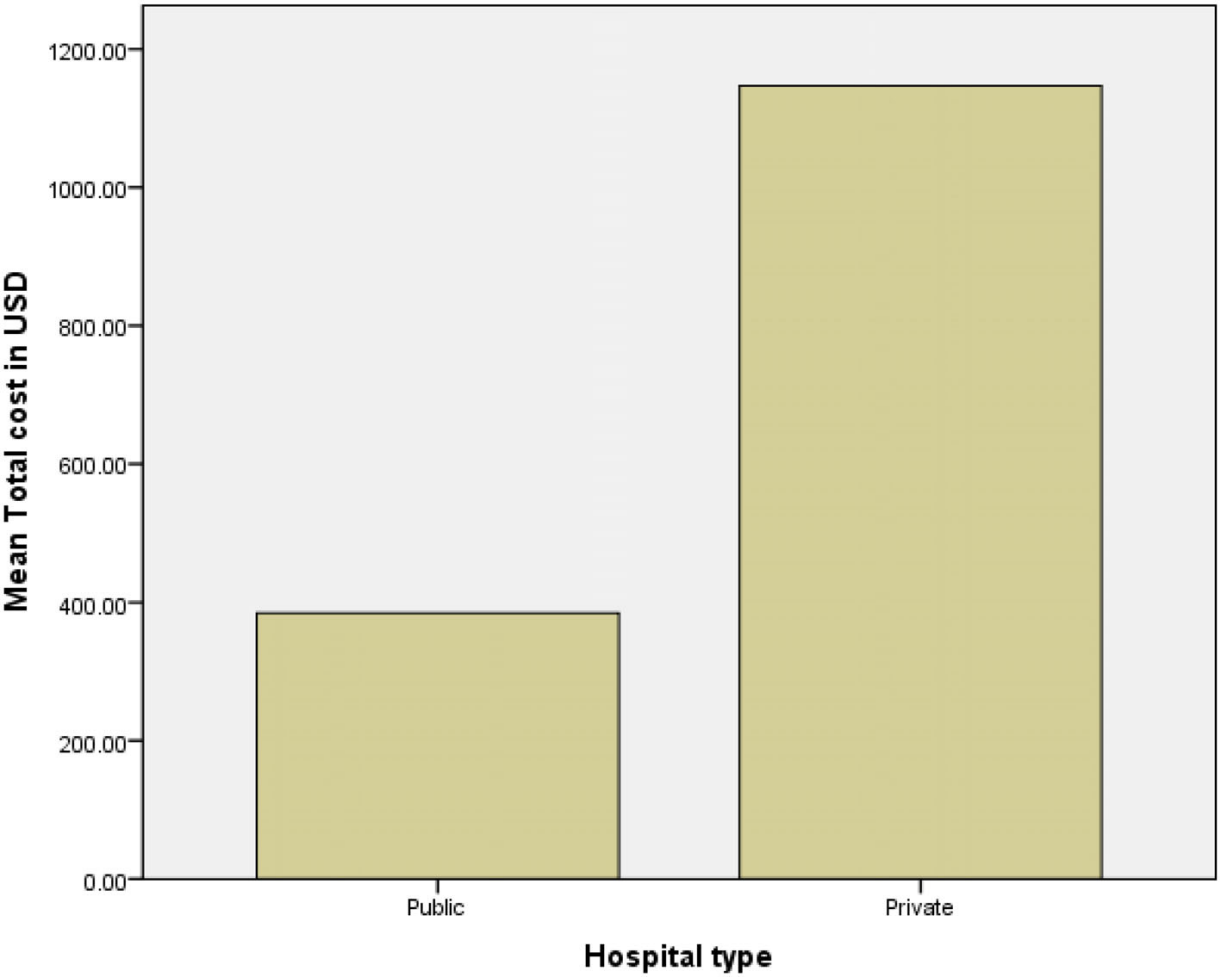
Illustration of the mean of the total cost as per patient with the type of hospital.

Figure 2 shows a bar chart that shows the mean total expenditure of the patients based on the type of hospital. It can be seen that, for a public hospital, the mean cost is 384.76 USD, whereas for a private hospital, the mean total cost is 1146.93 USD. It is evident that the private sector means three times the cost compared with the public sector.

### Cost of different segments for NAFLD

The commonest presentation of fatty liver disease is simple fatty liver/NAFLD, we have calculated the different segments of the cost of illness for NAFLD. The maximum cost was for investigations ranging from USD 9.39–375.59 (49.08%). The cost of medicines was next to this USD 0.09–783.85 (32.41%). Transportation cost was USD 0.94–300.44 (11.11%). The cost for the consultation of the physician was USD 0.28–9.39 (6.67%). The cost of food was USD 0.94–75.11 (5.25%). Tips had to be paid in the hospital was USD 0.00–96.94 (2.88%) (Table 3).

**Table 3:** Different expenditures for NAFLD

	USD	% (Mean ± SD)
Consultation fee	0.28–9.39	6.67 ± 9.15
Medicine cost	0.09–783.85	32.41 ± 18.48
Laboratory test	9.39–375.59	49.08 ± 36.33
Transportation cost	0.94–300.44	11.11 ± 13.83
Cost of food	0.94–75.11	5.25 ± 4.09
Informal payment/tips	0.00–96.94	2.88 ± 3.05
Total cost	16.90–955.08	

#### Liver transplantation

We could not estimate the cost of liver transplantation for NASH cirrhosis, because transplant facilities are not available in Bangladesh. We have communicated with two of our NASH cirrhosis patients who had been successfully transplanted in a neighboring country. The cost of transplantation was 61 027.13 and 65 721.53 USD.

## Discussion

In this study, besides baseline demographic data, direct medical costs such as hospitalization fees (IPD), consultation fees, laboratory investigation charges, purchases of drugs, and medical procedure expenses were estimated. Direct nonmedical costs like travel expenses and informal payments were considered. The minimum applicable costs were taken into consideration; hence it might be lower than the actual costs.

The mean age of the respondents was 43.78 ± 11.78 years. In a similar study, the mean age of the sample was 40.1 ± 9.5 years and females were more than males, which was 58.8% and 41.2%, respectively.^13^ In this study, with concern to body mass index, 3.3% were underweight, 35.5% were normal in weight, 48.8% were overweight, and lastly, 12% were in the obese category. Demographic characteristics are similar to previous studies,^3^, ^8^, ^9^, ^13^ which indicates that our study samples are representative of the general population.

For the cost analysis of all stages of diseases as well as comorbidities, differences in the cost of illness between public and private hospitals were statistically significantly higher in a private hospital. For NAFLD in public hospitals, the median cost was 138.95$ per patient, whereas in private hospitals, the amount was 190.87$. In addition, for NASH or NASH cirrhosis patients, the overall median costs were 2.63–3.2 times higher in private hospitals than that of the public hospital. In a previous study in Bangladesh, Begum et al. explored that expenditure in private hospitals was much higher than that of public hospitals.^13^, ^14^, ^15^, ^16^ Insufficient facilities at a public hospital to manage huge patients in OPD/IPD area are reasons for choosing private hospital. That is, the cause of progressively increasing “out of pocket expenditure” in Bangladesh reaching about 70%.^17^

This study illustrated that the cost per patient for NAFLD was 16.90–955.08 and NASH was 16.90–1032.54, whereas for NASH cirrhosis it amounted to 422.48–46 942)USD per patient in Bangladesh. In a study conducted in Hong Kong, Markov cohort state transition model with one‐year cycles and a 20‐year horizon to estimate the economic and clinical burden of NASH in the adult. NASH will cost $1.32 billion and 124 liver transplants over 20 years, with an average cost per person‐year of $257. In this context, they estimated that direct medical expenditure for NASH in 2017–2018 was $88 million, which is approximately 0.41% of the total health expenditure. This study also states that the average cost was $270 per person per year with the highest average cost per person‐year for patients aged 80+ ($297) and the lowest cost per person‐year for patients aged 50–59 ($264).^2^ Our expenditure/patient is lower than that of Hong Kong, but the total cost is much higher because of the huge population of Bangladesh.

In another study conducted in the United States, over 64 million people are projected to have NAFLD, with annual direct medical costs of about $103 billion ($1613 per patient).^6^ The cost of illness is much lower in Bangladesh than that in developed countries, for example, United States, Hong Kong, and Europe. Further reduction of healthcare expenditure is possible by increasing public healthcare facilities in Bangladesh.^18^

NAFLD is the most common form of fatty liver disease. We have addressed the cost of illness and the cost of different segments further for NAFLD, because at this stage different interventions will reduce the costs in the future. The cost/patient /visit (evaluation) was 157.91 USD in this study for NAFLD. As 45 million people are suffering from fatty liver in the country, the total cost for a single evaluation of all patients will be 7.11 billion USD. This cost is one‐tenth of the annual national budget of 72 billion USD for 2022–2023.^20^ The annual health budget of Bangladesh for 2022–2023 is 3.46 billion USD,^21^ so this is double the annual health budget. In this study, few patients of NAFLD had minimum expenses regarding medicine and consultation fees. No expense in medicines, at least for a significant number of patients of NAFLD will be reasonable as there is no recommendation of specific medicine for the management of NAFLD. Although the cost of medicine was 0.09–783.85 (32.41%) for NAFLD in this study, preparing a national guideline for the management of NAFLD and training primary care physicians will decrease the expenditure on medicine in the future. This will also decrease the transportation costs for the unnecessary rush to a specialty center. A referral pathway may also reduce the cost of consultation fees and transportation, consultation fees, and food costs.^19^ In this study, transportation costs per person were 0.66–300.44 USD.

As in the United States Japan, Europe, the United Kingdom, Iran, the Middle East, and other developed and developing countries, the burden of fatty liver certainly is increased in future.^4^, ^7^, ^22^, ^23^, ^24^, ^25^, ^26^, ^27^, ^28^, ^29^, ^30^ The study from France, Germany, Italy, Spain, the United Kingdom, and the United States in 2018 found that the mean total cost/per patient/year for NASH was €2763, €4917, and €5509 for direct medical, direct nonmedical, and indirect costs, respectively.^23^

Another study included five countries in Europe that estimated total economic costs were €8548–19 546 M. Of these, health system costs were €619–1292 M, and total well‐being costs were €41 536–90 379 M.^4^ This study is similar to the current study in increasing cost with the severity of the disease. But the per person cost is much higher than that of Bangladesh. A study from Japan explored that the mean total all‐cause healthcare costs for NAFLD ranged from ¥322 206 to ¥340 399 per patient per year between 2011 and 2017.^24^ Cost increased with increasing comorbidities. The current study found a similar trend of increasing costs for diabetes and hypertension with NAFLD.

By 2030, in the KSA, UAE, and Kuwait, NAFLD cases are estimated to double, leading to costs of NASH standard care increase totaling USD40.41 bn, 1.59 bn, and 6.36 bn, respectively. But in 2019, NASH‐related costs comprised, respectively, 5.83%, 5.80%, and 7.66% of national healthcare spending.^30^ The same prediction of the trend of prevalence of NAFLD and increasing expenditure may be projected for Bangladesh.

The current trend diagnosis of NASH by histopathology and the nomenclature of NAFLD by excluding many relevant diseases may be the cause of increasing costs. In this study, the maximum cost was for investigations. This might be reduced by applying the new nomenclature of metabolic‐associated fatty liver disease (MAFLD), where the diagnosis is of inclusive character rather than that of exclusive character.^31^

The study did have certain limitations. Only a brief treatment period was considered. All direct and indirect costs were not included. In some cases, the follow‐up process may have been excluded, as the diseases are progressive. Underestimations of costs are there as the minimum applicable costs were considered. The cost of liver transplantation was not estimated in this study. Intangible costs cannot be calculated or estimated. Despite the limitations, the results are valuable as they illuminate the cost of NAFLD illness in Bangladesh that was never addressed before.

## Conclusion

This study revealed the huge cost of illness for NAFLD. It is concerning that the cost is highest for end‐stage liver disease and that comorbidities with diabetes and hypertension can further increase the cost. It is encouraging, however, that preventive measures, training of primary care physicians, referral pathways, changing the nomenclature of NAFLD to MAFLD, and increasing facilities at the public hospital and national guidelines could potentially reduce the cost of illness in the future. Policymakers, clinicians, and every stakeholder should pay attention to the burgeoning clinical and economic burden of NAFLD.

## Funding statement

Funded by the Research grant from Bangabandhu Sheikh Mujib Medical University. Funding number: 211/2021.

## Ethics approval statement

This research was approved by the Institutional Review Board (IRB) of Bangabandhu Sheikh Mujib Medical University.

## Patient consent statement

Informed written consent was obtained from every participant.

## Permission to reproduce material from other sources

Not related.

## Data Availability

Data will be available on request.
